# An Exploration of Attitudes toward Dogs among College Students in Bangalore, India

**DOI:** 10.3390/ani9080514

**Published:** 2019-07-31

**Authors:** Shelly Volsche, Miriam Mohan, Peter B. Gray, Madhavi Rangaswamy

**Affiliations:** 1 Boise State University, Boise, ID 83725, USA; 2 CHRIST (Deemed to be University), Bengaluru, Karnataka 560029, India; 3 University of Nevada, Las Vegas, Las Vegas, NV 89154, USA

**Keywords:** India, dogs, anthrozoology, human-animal interactions, companion animals, street dogs, attitudes, shared spaces, urban dogs, pet dogs

## Abstract

**Simple Summary:**

The presence of dogs in urban spaces and family homes is becoming increasingly common worldwide. Despite this, investigations into cultural variations in this practice are still new. Using self-report, pen-and-paper surveys, we explored attitudes toward both pet dogs and stray dogs in an urban, college sample in Bangalore, India. We found a notable presence of pet dogs in homes or desire to have a pet dog, as well as the use of affiliative terms (family, companion) regarding these pets. Not surprisingly, we also found expected sex differences between men’s and women’s attitudes toward pet dogs and stray dogs in shared, urban spaces.

**Abstract:**

Conversations in the field of anthrozoology include treatment and distinction of food animals, animals as workers versus pests, and most recently, emerging pet trends including the practice of pet parenting. This paper explores attitudes toward pet dogs in the shared social space of urban India. The data include 375 pen-and-paper surveys from students at CHRIST (Deemed to be University) in Bangalore, India. Reflecting upon Serpell’s biaxial concept of dogs as a relationship of *affect* and *utility*, the paper considers the growing trend of pet dog keeping in urban spaces and the increased use of affiliative words to describe these relationships. The paper also explores potential sex differences in attitudes towards pet and stray dogs. Ultimately, these findings suggest that the presence of and affiliation with pet dogs, with reduced *utility* and increased *affect*, is symptomatic of cultural changes typical of societies encountering the second demographic transition. Despite this, sex differences as expected based upon evolutionary principles, remain present, with women more likely to emphasize health and welfare and men more likely to emphasize bravery and risk taking.

## 1. Introduction

Despite its ubiquity, the role of “the dog” varies with social context. In 2011, Gray and Young [1] found that dogs are the most frequently kept “pet” cross-culturally. However, the dogs in their data often served functions more akin to hunting aids, pest control, home sentinels, and even food. These utilitarian roles are distinct from the Oxford Living Dictionaries’ definition of pet: “a domesticated or tamed animal kept for companionship or pleasure” [2]. In some cases, dogs may even be viewed as pests that carry disease [3,4]. This is the case in many parts of India, where research on human-dog interactions often focuses on the relationships between dog bites, disease, and the view of domestic dogs as an invasive species [5,6].

This view is changing in many parts of the world, particularly in post-transition societies. Lesthaege [7] identified the Second Demographic Transition (SDT) as a time in which specialization, opportunity, and transitions from the industrial complex would afford individuals increased autonomy. The SDT is marked by economic growth, decreased total fertility, increased educational attainment, and the choice for new types of household. For example, in the United States, arguably well past its SDT, women’s educational and economic attainment continue to be on the rise while fertility rates have fallen [8]. It has also been observed that these families who do not have children, whether by choice or circumstance, often choose to apply their parenting strategies to pets, especially dogs [9,10,11,12].

**Figure 1:**
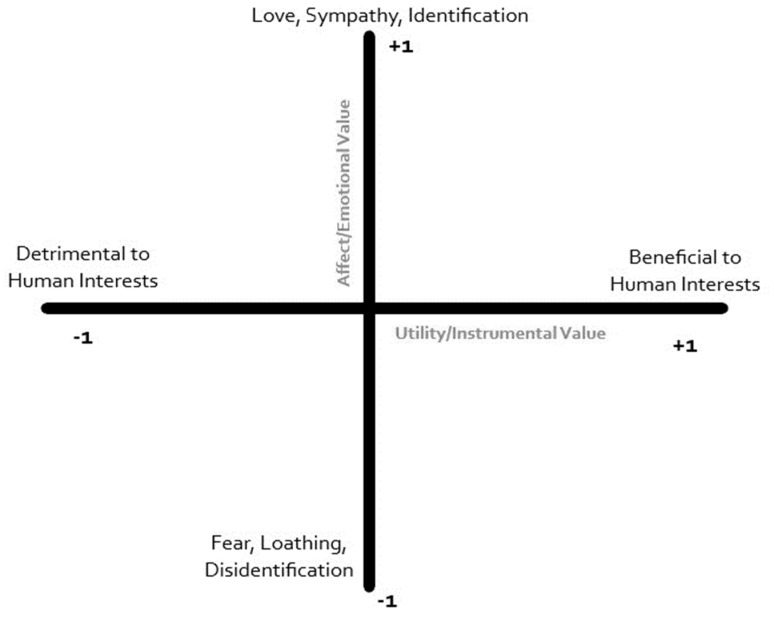
Serpell’s *affect* and *utility* biaxial model.

To understand this wide range of attitudes toward domestic dog, Serpell [13] proposes a biaxial model. He suggests that whether viewing individual dogs or the cultural archetype “the dog”, dogs exist on two continuous axes that intersect and interact (see Figure 1). On one axis is the *utility* of dogs, ranging from detrimental to beneficial to human interests. Beneficial *utility* includes roles such as those found by Gray and Young [1], while detrimental *utility* includes disease carriers and dogs who are a potential danger to the humans with whom they share space. The other axis considers the *affect* related to dogs, ranging from fear and loathing to love and identification. The *affect* axis may be viewed as encompassing measurable characteristics such as attachment, appreciation for the symbolism of the animal, or the willingness to sympathize [14].

While there are instances of cultures who may be to the extreme on one or both axes, most ideologies exist in a nuanced space elsewhere in the resulting quadrants. This complexity modifies a person’s attitudes toward dogs based upon prior experiences [14]. For example, a dog may be viewed with moderate *affect* as a result of its usefulness (high *utility*) in the family’s mode of production. In other cases, fear of harm (negative *affect*) may be the result of low *utility* or a high probability of dangerous interactions. As dogs move further up the scale in *affect*, they often become neither positive nor negative in *utility* [14,15]. In these instances, dogs become well-loved but neither help nor harm the owner. In extreme cases, attachment may become detrimental on the *utility* scale, causing owners to incur negative life impacts because of prioritizing their dog’s needs. Educational opportunities, global movements toward animal welfare, and growing global perceptions of animals as autonomous agents in need of protection from exploitation often contribute to these changes [15].

As societies move through the SDT, a trend exists toward urban centers that are less family friendly [16] and generates a need to investigate the role of non-human animals at multiple social levels, beyond “add non-human animals and stir” [17]. Rather, there is an increasing need to consider non-human animals as social actors with whom humans negotiate space and relationships. Based upon our observations of where, and when, pet keeping and pet parenting practices develop, the SDT serves as a catalyst to move pet dogs into the higher *affect*, moderate *utility* quadrant of Serpell’s model. South Korea [18], Japan [19], and China [20] are each showing signs of embracing pet dogs in this way.

As part of investigating these changing attitudes toward dogs, a focus on human sex differences becomes relevant. Herzog [21] found that women tend to be more likely to show positive attitudes toward animals, such as protectionism and a focus on wellbeing. In contrast, men in his review were more likely to engage in hunting and hold less favorable attitudes toward welfare efforts. On one hand, this makes sense given women’s increased sensitivity to risk in contrast to men’s higher frequency of risk-taking [22]. However, Blazina and Kogan [23] found that young men’s risk-taking with dogs and lack of empathy may also be due to increased awareness of social expectations connected to norms of masculinity.

### 1.1. Dogs in India

Despite the amount of prior work, little research has been done to explore if and how these changes are present in India. A colonial history has left an imprint on modern relationships between Indians and dogs. There are mixed reactions among people and the government regarding how to handle strays in urban areas, and much of the concern is driven by fear of disease such as rabies [24]. Initially, stray populations were controlled by collecting unowned dogs and euthanizing those who were unfit for adoption. More recently, pressures from animal welfare organizations have resulted in animal birth control practices based upon trapping, neutering, and releasing or adopting strays. This practice is supported by certified veterinary schools and trained and certified members of society, many of whom also become certified in handling dogs and feeding colonies [25].

Most related scholarly work in India continues to focus on free-ranging dogs. This research finds that dogs in rural Indian villages may belong to the community as shared dogs [26] or are relegated to the edges of the village as pariahs [27]. Often, this work emphasizes the role free-ranging dogs play in disease vectors, particularly for rabies [28] or as invasive species on local wildlife [5]. This is likely due to the large population of free-ranging dogs in many parts of India [29], including both rural villages and urban centers. Social spaces are often shared, and despite disease concerns, some researchers argue that free-ranging dogs are an important part of the urban ecology [30].

Amidst this focus on shared spaces and free-ranging dogs, the practice of pet dog keeping is growing sufficiently to support a burgeoning market for foods, veterinary care, and other supplies and resources that experts estimate to top $270 million USD by 2019 [31]. Given that a contrast between urban and rural ideals toward free-ranging dogs has been observed in societies with similar historical backgrounds [32], it seems likely that the SDT in urban centers changes perceptions of and sympathy toward free-ranging dogs. Despite the lack of comparative work between urban and rural areas done on India’s dog attitudes, we argue that if the SDT does influence a society’s *affect* toward “the dog,” we should be able to capture a positive *affect* toward dogs in a sample of educated, urban young adults.

The aims of this exploratory study were to (a) investigate current attitudes among an urban, college cohort in India regarding pet and free-ranging dogs; (b) determine what, if any, sex differences exist between the attitudes of male and female respondents; and (c) determine what, if any, perceived differences exist between participants and their elders (i.e., parents, grandparents). We chose a convenience sample of urban, college students in Bangalore, India, and included our survey questions as part of a larger project probing the role of grandparents in college students’ lives.

## 2. Materials and Methods

### 2.1. Participants and Procedures

We recruited respondents aged 17- to 25-years of age from the undergraduate and graduate student population at Christ (deemed to be University) in Bangalore, India. Flyers were posted on campus with information for interested individuals to respond. While completing the Informed Consent, individuals were asked to confirm their student status, age, and lack of children. Upon completion of the consent forms and the survey, respondents were offered a coupon to exchange for breakfast and coffee/tea.

Recruitment and data collection began only after approval of appropriate ethics review boards at both institutions. The study was deemed exempt by Social/Behavioral IRB at University of Nevada, Las Vegas and CHRIST (Deemed to be University) committee reviewed the proposal and related documents, then issued an approval certificate on the basis of little or no harm and complete compliance with the institution’s ethical rules. A letter attesting approval to collect data on campus was received from the Student Affairs Director as well.

### 2.2. Questionnaire Design

We utilized a paper-and-pencil questionnaire format written in English, as it was determined that English is the predominant language spoken by students. The survey included general demographic questions (e.g., age, gender, and socioeconomic status). The Kuppuswamy scale uses education level, profession, and income of the head of household to compute a score that indicates socioeconomic status levels [33]. We did not ask any questions regarding religion given the sensitivity of the area; however, it was noted by the coauthors that the university includes many students of non-Christian backgrounds.

Additional questions regarded current and future dog ownership (See Appendix A). If individuals answered yes to “Do you have a pet dog?”, an additional subset of questions sought to better understand the perspective of owned dogs. These open-ended questions included “where does it sleep?”; “Is your dog tied, chained, or otherwise confined to your home?”; “What do you regularly feed it?”; “Does it have a name?”; and “How do you demonstrate affection for your pet dog?”. The question “How do others in your family treat or interact with your pet dog?” sought to understand variations in generation and gendered ideals regarding pet dogs. Related questions sought to understand family involvement and attitudes toward dogs. For example, “Do you have conversations with your family regarding (pet or stray) dogs?” with the options “Yes” or “No” was asked for pet dogs and stray dogs separately. Likewise, “How do your attitudes toward (pet or stray) dogs compare with your elders (mother, father, grandparents)?” with the options “same/similar,” “more positive,” “more negative,” and “can’t say” was asked for pet dogs and stray dogs separately.

Further questions investigated broader attitudes toward dogs, regardless of current ownership. Responses to “What is the main purpose of having a pet dog?” included “pet,” “playmate,” “companion,” “family,” “protector,” and “worker.” Additionally, a space for “other (please explain)” was provided to capture perspectives that may have been missed during creation of the survey. Respondents were also asked whether they hope to have a dog in the future or whether they would prefer a pedigree dog, Indie (local stray) dog, or would adopt either. Finally, open-ended questions included “Is there anything else you would like to share with us regarding your views on pet dogs?” and “Is there anything else you would like to share with us regarding your views on stray dogs?” gave respondents opportunities to expand upon items or provide information they felt was not otherwise covered.

Finally, two lists of statements sought to understand attitudes toward pet dogs and stray dogs. Responses were measured on a 5-point Likert scale ranging from “1 = Strongly Agree” to “5 = Strongly Disagree.” The pet dog list included statements such as “Regular veterinary care is important for pet dogs” and “Pet dogs should be trained to behave properly.” The stray/feral dog list included statements such as “I am afraid of dogs on the street” and “I interact with stray/feral dogs.” Though initially included to provide a comparison of attitudes toward pet dogs and street dogs, ultimately these statements proved more useful in comparing gender differences in respondents’ perceptions and behavior. The questions were originally written with common attitudes toward pet or stray dogs, respectively, in mind. As such, we acknowledge the questions may appear biased or leading. Despite this, as noted in the Results section, clear sex differences were present in our sample.

### 2.3. Analysis

Upon collection of surveys, answers were entered in Microsoft Excel and numerically coded as appropriate, then transferred to IBM SPSS V.24 for analysis. All respondents were given the option to leave blank any questions they were uncomfortable or uncertain answering. While these data would normally be removed from the sample, the unanswered questions were sporadic in nature. Removing them would result in a significantly smaller sample size on the most pertinent questions. As such, we report the total number of “no answer” in the results when appropriate. Demographic information is reported in terms of percentages and means. We used a Mann-Whitney *U*-test to analyze sex differences in the Likert scale questions regarding attitudes toward pet or stray dogs. Finally, any open-ended questions were reviewed using a standard thematics approach.

## 3. Results

A total of 375 surveys were completed. Respondents reported a mean age of 20.37 years (SD = 1.82). A total of 129 (34.4%) men and 243 (64.8%) women responded, while 3 (0.8%) individuals did not report their gender. While socioeconomic status ranged from lower class to upper class, the population was heavily skewed toward upper (62.1%) and upper middle (26.9%) class. A total of 95 (25.3%) respondents currently own a dog, while 179 (47.7%) respondents reported wanting a dog in the future (See Table 1 for demographic data). Of the 293 individuals who answered the question regarding breed preference, 62.1% stated they preferred a pedigree/purebred dog, 19.1% preferred an Indie (local stray) dog, and 18.8% either circled both or responded by writing “either.”

One-hundred-thirty-four individuals did not answer the question “What is the main purpose of having a pet dog?”. Additionally, despite the wording of the question asking for the “main purpose”, there was no specification made regarding the number of options that could be chosen. As such, many respondents selected multiple options for this question. In these instances, we coded the response based upon the most affective option selected. (For example, if both “family” and “pet” were chosen, the answer was coded as “family.”) This resulted in the following breakdown: “pet” (7.5%), “playmate” (7.9%), “companion” (42.7%), “family” (24.5%), “protector” (8.3%), and “other” (9.1%). When selecting “other,” respondents were encouraged to explain, with answers ranging from “slave” and “status symbol” to “best friend” and “helper.

When asked whether they talk to their families about pet or stray dogs, the majority of respondents reported yes, with no statistically significant difference between the type of dog discussed (59.7% report discussing pet dogs while 57.9% report discussing stray dogs). Relatedly, respondents were asked about their attitudes toward pet dogs and stray dogs, separately, when compared with their elders. Response options included “same/similar,” “more positive,” “more negative,” and “can’t say.” Most respondents stated they have “more positive” attitudes towards pet dogs (33.9%) and stray dogs (30.9%), some stated having “same/similar” attitudes toward pet dogs (23.5%) and stray dogs (28.5%), and the fewest answered “more negative” (8.3% for pet dogs, 12.3% for stray dogs). This suggests that urban, Indian college students are talking to their families about both pet dogs and stray dogs, and there may be a change across generations in the perception of dogs. We unpack this further in the Discussion.

We utilized a grounded theory and thematic approach to explore themes within responses to the open-ended questions regarding current dog ownership. When asked “how do you demonstrate affection for your pet dog?” the most common answers involved forms of kissing, cuddling, and hugging (29 out of 95 respondents), petting and grooming (26 out 95 respondents), and direct interaction like play and talking (29 out of 95 respondents). Only nine of 95 respondents reported feeding their dogs or using treats or food as a show of affection.

In response to “where does it sleep?” nearly half of the respondents stated a sleeping place within the house. Of those, 28 specifically mentioned their dog sleeps with humans on furniture, like a couch, or in bed with members of the family. Eighteen respondents reported that the dog sleeps in a cage or kennel but did not specify if these spaces were inside the house or outside the house. Thirteen respondents specified that the dog sleeps outside, on the veranda, in the yard, or a car port. Given India’s dichotomous relationship with dogs between urban and rural areas, it is also noteworthy that three individuals mentioned not having dogs in the city, but that they had dogs “in (their) hometown” or “at home, where we take care of the village dogs.”

When asked about what they regularly feed their dogs, respondents were split between traditional dog food (kibble types), home cooking, or a combination thereof. The most common home cooked foods included chicken, roti or rice, and potatoes. Other forms of meat were occasionally mentioned, and eggs and milk were also documented. Highlighting the range of attention and education regarding canine nutrition, variations of this home cooking and kibble combination spanned from one respondent to specifically mentioned giving their dog additional supplements to others who stated the dog ate “whatever it wants” or “whatever the family is eating.”

Finally, Mann-Whitney *U* tests found significant sex differences in responses to the 5-point Likert scale questions on attitudes toward pet dogs and stray/feral dogs. Overall, female respondents were significantly more likely to agree with statements regarding the health and welfare of dogs, such as “Pet dogs should receive regular vaccinations” (*U* = 9670.00, *p* = 0.001) and “Pet dogs need regular exercise to stay healthy” (*U* = 9979.00, *p* = 0.006). Males were significantly more likely to agree with statements that display a lack of fear. For example, males were more likely to agree with the statement “I interact with stray/feral dogs” (*U* = 12626.50, *p* = 0.023) and more likely to disagree with the statement “I am afraid of dogs on the street” (*U* = 13016.00, *p* = 0.038). Interestingly, there appear to be agreements between males and females on statements related to humane education and media such as “Pet dogs should be spayed or neutered to control the population of unwanted dogs” (*U* = 10931.00, *p* = 0.156) or “Stray/feral dogs should be cleared from public spaces and streets” (*U* = 14376.00, *p* = 0.642). A complete list of statements, *U* scores, and *p*-values can be found in Table 2.

## 4. Discussion

In this study, we utilized a paper-and-pencil questionnaire format that included questions related to demographics (e.g., age, gender, socioeconomic status), current and future dog ownership, attitudes toward pet and stray dogs, and perceptions of attitudes compared to other, older family members (parents, grandparents). The results suggest that our sample of urban, Indian college students exhibit attitudes of positive *affect* toward pet dogs and practices in pet keeping that are like many other cultures entering or experiencing the Second Demographic Transition. This is particularly noted in questions regarding pet dog keeping practices among the respondents who currently own dogs, the large number of respondents who report hoping to own dogs in the future, and the reported value of dogs as family and companions.

It is important to highlight the words identifying the role of pet dogs. Over 50% of respondents identified dogs as “family” or “companion.” In a culture often researched for its struggles with free-ranging dogs, it is noteworthy that dogs in respondents’ urban homes are identified with terms suggesting human-like roles. This mirrors findings in a United States study in which women identified their relationships with pet dogs using similarly familial terms such as “guardian” or “parent” [10]. While these terms do not suggest identical levels of affinity, it is noteworthy that most are terms that would be used to relate to persons as opposed to beasts of burden. Likewise, Blouin [34] found that individuals often identify dogs as child surrogates or companions as opposed to workers or pests when perceiving dogs with a humanistic or protectionistic attitude. This transition in the role identification of pet dogs appears to be emerging in various cultures worldwide.

This is reflected in the nearly 50% of respondents who reported wanting a pet dog in the future, as well as the ways in which current pet dog owners in this sample display affection. Petting, kissing, hugging, and cuddling were among the most common forms of expression reported among those who currently owned pet dogs. Likewise, play and talking to pet dogs was also mentioned repeatedly. These displays of affection mirror what is commonly seen in post SDT media (i.e., movies, social media posts), advertisements, and regularly at pet related events [35]. Given that most respondents reported a “more positive” or “same/similar”, it stands to reason that a trend toward this type of attached, increased *affect* pet dog keeping is likely to continue.

Similarly, the feeding practices of respondents who own dogs are noteworthy. Most respondents either feed commercial kibble, home cooking, or a combination thereof. While it is possible that these feeding practices come from a lack of spending on pet dogs in the home (i.e., feeding whatever is available), this is unlikely the case given the socioeconomic status of the respondents and their families. Rather, it is more likely that these individuals are attempting to engage in quality care of the pet dogs in their homes, contributing to the nearly $270 million USD pet products industry in India [31].

It appears that changes to our relationships with pet dogs can be viewed as indicative of broader cultural changes. Using Serpell’s [13] biaxial model, environments in which pet dogs move into the high *affect*, moderate or zero *utility* quadrant are often those on the edge of or already well into their second demographic transition (SDT). Education rates are high, traditional industry is giving way to technology and specialization, and fertility rates are low [7,8]. India is likely undergoing the SDT in many urban areas. As early as the 1960s and 1970s, Goode [36] reported that India was already seeing a transition from the joint family unit to a nuclear family consisting predominantly of a married couple and their children. Singh [37] provides evidence that the nuclear family has arrived as the dominant form of familial residence, particularly in urban centers where education and occupational specialization thrive. If the SDT in India follows trends seen in other cultures [7,8,11,13,18,19,20], the attitudes our respondents displayed toward pet dogs may suggest that upcoming generations may preference pets and personal authenticity over familial expectations of marriage and large families, at least in the higher socioeconomic groups.

This may be particularly true as more females attend university and seek professional careers. Repeatedly, a connection is found between female reproduction and educational attainment. Looking at women’s reproduction across socioeconomic and educational status in India, Bhat [38] found that fertility delays and declines were not only connected to an individual female’s education. Rather, diffusion of contraceptive practices to parents resulted in reduced fertility among illiterate women who chose to emphasize educational attainment for their daughters. This further suppression of fertility and continued delay of marriage often intersects with individuals who choose to engage in high *affect*, moderate or zero *utility* relationships with pet dogs as family or surrogate children [9,10,11].

For this reason, it is also interesting to see that evolutionarily expected sex differences appear in attitudes toward pet dogs and stray/feral dogs. Since it is unlikely that a large percentage of any age cohort will forego parenthood, it makes sense that female respondents were significantly more likely to agree with statements of caretaking, health, and welfare (for example, “Pet dogs should be groomed regularly”) while male participants are more likely to agree with statements that suggest bravery or a lack of fear (for example, “I interact with stray/feral dogs”). A systematic review completed by Archer [39] found that men are more likely to participate in risk-taking, competitive behaviors, while women are more skilled in social domains and are more likely to display empathy. Likewise, literature on parenthood consistently supports that women are more involved in hand’s on, direct care [40,41] which often translates toward sex differences in care toward other species.

As the first species domesticated by humans, *Canis familiaris* has been with us for at least 15,000 years [4]. This extensive history together has seen the human-canine bond evolve from one of primarily low *affect* and high *utility* to one that is becoming increasingly high *affect* and moderate or zero *utility*. While variation continues to remain, it appears that the domestic dog is continuing to move further into our homes [15]. Global influences and technology are pushing urban centers into the Second Demographic Transition, with a growing emphasis on personal development that results in multiple shifts in the practice of family. These changes involve reduced fertility, delayed marriage, and the inclusion of non-human animals in the family—especially the dog. Based upon the current study and related literature, we suggest that it is time to view human-dog relationships as a symptom of major social changes resulting from these trends.

## 5. Conclusions

Further research is needed to determine whether the results of this study remain for other populations within India and other cultures. Similarly, ethnographic observations and interviews may serve to provide clarification between the reported ideals and the daily practice of human-dog relationships. Nonetheless, the literature continues to grow, and more data suggest that there is a transition occurring in the practice of the bond between humans and “human’s best friend.”

As is always the case, there are limitations to this study. To begin, all surveys are self-reported, remaining open to the intentional or unconscious bias of the respondents. This may add to or be the result of expectations related to the skewed socioeconomic status of the respondents toward upper-middle and upper class. Relatedly, the actual daily practices of pet dog keeping in India may not be reflected in these data, given that not all members of the population, urban or rural, have the same access to resources to care for dogs in the same manner.

Likewise, only one generation was surveyed, which may lead to irregularities in the actual versus perceived differences regarding attitudes toward pet dogs. To avoid potential biases, it would be relevant to repeat the study and attempt to target both or multiple generations to determine whether this trend continues to hold. However, it is worth noting that a comparison of existing literature with the results of this survey suggest that the reported differences in attitudes toward pet dogs would remain.

Finally, while much of the literature on the growing trend toward affective pet keeping describes the trend in positive light, our collective personal experiences working with pet owners, volunteering in animal welfare programs, and speaking to other researchers suggests another side needs investigation. Future work should seek to understand why animal abandonment happens while considering these reported attitudes. Though we surveyed urban, college students, many of whom own dogs, this does not negate the large population of free-ranging dogs in India and other parts of the world. Likewise, if humans are embracing pets as family, a deeper understanding is needed of how and why these animals end up relinquished in shelters or abandoned on the street.

Despite these limitations, the current research adds to a growing body of literature that, when combined, suggests a trending change in how humans are interacting with their pet dogs. As shifts in this relationship occurred in our past when food became fellow hunters and fellow hunters became home protection, we argue that watching the many ways in which the human-dog bond responds to social changes across the globe may provide light on human trends in urban living and kinship.

## Figures and Tables

**Table 1:** Demographic data of survey respondents.

Gender	*n* (%)
Female	243 (64.8%)
Male	129 (34.4%)
No Answer	3 (0.8%)
Socioeconomic Status	
Upper Class	233 (62.1%)
Upper Middle Class	101 (26.9%)
Lower Middle Class	16 (4.3%)
Upper Lower Class	5 (1.3%)
Lower Class	1 (0.3%)
No Answer	19 (5.1%)
Age	
Range = 17–25 years, µ = 20.36, SD = 1.82	
Current Dog Ownership	
Yes	95 (25.3%)
No	261 (69.6%)
No Answer	19 (5.1%)
Desire for Future Dog Ownership	
Yes	179 (47.7%)
No	69 (18.4%)
No Answer	127 (33.9%)
Dog Breed Preference (Purbred vs. Local/Indie) *	
Pedigree/Purebred	182 (62.1%)
Indie/Local Stray	56 (19.1%)
Either	55 (18.8%)
Purpose of Dog **	
Pet	18 (7.5%)
Playmate	19 (7.9%)
Companion	103 (42.7%)
Family	59 (24.5%)
Protector	20 (8.3%)
Other	22 (9.1%)

* 82 respondents did not answer the question on dog breed preference. Reported percentages are based upon the remaining 299 who did. ** 134 respondents did not answer the question on a dog’s purpose. Reported percentages are based upon the remaining 241 who did.

**Table 2:** Likert scaled statements and associated *p*-values for sex differences.

	µ Rank *: Men	µ Rank *: Women	Mann-Whitney *U*	# of Responses **		*p*-Value
**Statements regarding perceptions of pet dogs**				*Men*	*Women*	
Regular veterinary care is important for pet dogs.	188.15	155.48	9895.00	112	220	0.001
Pet dogs should receive regular vaccinations.	189.16	154.16	9670.00	112	219	0.001
Pet dogs should be spayed or neutered to control the population of unwanted dogs.	154.48	169.63	10931.00	111	217	0.156
Pet dogs should be groomed (i.e., bathed, nails clipped, oral hygiene) regularly.	195.47	150.93	8963.50	112	219	0.001
Pet dogs need regular exercise to stay healthy.	182.78	155.28	9979.00	110	218	0.006
Pet dogs should be trained to behave properly.	187.14	150.65	9170.00	110	215	0.001
**Statements regarding perceptions of stray/feral dogs**						
Dogs are dangerous.	194.98	172.80	12877.50	125	235	0.045
Dogs in packs are dangerous.	192.51	174.90	13311.50	125	236	0.117
I am afraid of dogs on the street.	197.20	173.92	13016.00	126	237	0.038
Dogs are dirty animals.	186.53	179.59	14360.50	126	237	0.529
I interact with stray/feral dogs.	164.01	189.27	12626.50	125	235	0.023
The sound of dogs barking is irritating.	168.39	188.50	13216.50	126	236	0.072
Stray/feral dogs should be cleared from public spaces and streets.	184.40	179.17	14376.00	126	235	0.642
Stray/feral dogs are the concern of civic bodies/non-governmental organizations/communities/neighborhoods.	159.13	157.33	11451.00	117	198	0.861

* Mean rank scores are listed for both male and female respondents. Lower scores suggest more agreement with the statement (based upon Likert scale ranks “1=Strongly Agree” to “5=Strongly Disagree.” ** The total responses did not vary greatly between men and women. However, approximately 30 respondents who did not currently own a pet dog did not complete the questions regarding attitudes toward pet dogs.

## Appendix A

List of questions included in the survey beyond demographics.
Questions regarding pet dog ownership: Do you have a pet dog? Yes/No If yes, please answer the following questions: How many dogs? Age of your dog(s) __________ Where does it sleep? Is your dog (1) tied, (2) chained, or (3) otherwise confined to your home? Is your dog allowed to roam the streets freely? What do you regularly feed it? How do you demonstrate affection for your pet dog? How do others in your family treat or interact with your pet dog? What is the main purpose of having a pet dog? (select only one) Pet Playmate Companion Family Protector Worker Other (specify in a word) If you do not have a pet dog, would you like one in the future? Yes/No Which type would you prefer as a pet? (a) Indie (local stray) dog or (b) pedigree dog Do you have conversations with your family regarding pet dogs? Yes/No How does your attitude toward pet dogs compare with your elders (mother, father, grandparents)? same/similar more positive more negative can’t say
Please answer the following questions regarding your perception of pet dogs. (5-point Likert Scale) Regular veterinary care is important for pet dogs. Pet dogs should receive regular vaccinations. Pet dogs should be spayed or neutered to control the population of unwanted dogs. Pet dogs should be groomed (e.g., bathed, nails clipped, oral hygiene) regularly. Pet dogs need regular exercise to stay healthy. Pet dogs should be trained to behave properly. Please answer the following questions regarding your perception of stray/feral dogs. (5-point Likert Scale) Dogs are dangerous. Dogs in packs are dangerous. I am afraid of dogs on the street. Dogs are dirty animals. I interact with stray/feral dogs. The sound of dogs barking is irritating. Stray/feral dogs should be cleared from public spaces and streets. Stray/feral dogs are the concern of civic bodies/non-governmental organizations/ communities/neighborhoods. Do you have conversations with your family regarding stray dogs? Yes/No How do your attitudes toward stray dogs compare with your elders (mother, father, grandparents)? same/similar more positive more negative can’t say
What is your biggest concern regarding stray dog(s)? Is there anything else you would like to share regarding your views on pet dogs? Is there anything else you would like to share regarding your views on stray dogs?
